# Risk perceptions of drinking bottled vs. tap water in a low-income community on the US-Mexico Border

**DOI:** 10.1186/s12889-022-14109-5

**Published:** 2022-09-09

**Authors:** Kerton R. Victory, Amanda M. Wilson, Nolan L. Cabrera, Daniela Larson, Kelly A. Reynolds, Joyce Latura, Paloma I. Beamer

**Affiliations:** 1 Department of Community, Environment, & Policy, Mel and Enid Zuckerman College of Public Health, University of Arizona, Tucson, USA; 2 Department of Educational Policy Studies and Practice, College of Education, University of Arizona, Tucson, USA; 3 Mariposa Community Health Center, Nogales, USA

**Keywords:** Border health, Water quality, Water consumption, Latinos, Bottled water, Tap water

## Abstract

**Background:**

Previous studies have shown that low-income Latinos generally drink bottled water over tap water and might be at increased risks for cavities from unfluoridated bottled water. In order to better design interventions, it is important to understand the risk perceptions of this unique high-risk yet historically marginalized group.

**Methods:**

We interviewed low-income Latino households (*n* = 90) from Nogales, Arizona who primarily drink bottled water and asked them to evaluate potential health risks of drinking tap water compared to 16 other voluntary activities. Unpaired t-tests were used to determine if statistically significant (α = 0.05) differences occurred in perceived risk by drinking-water source and differences among demographic groups in their level of (dis)agreement with statements regarding tap or bottled water safety. To assess significant differences (α = 0.05) in perceived risks and voluntariness to engage in a number of activities, including drinking local tap water and drinking water in different geographic regions, a one-way analysis of variance (ANOVA) followed by Scheffe’s post-hoc test (a conservative post-hoc test) with adjustment for the number of pairwise comparisons was used.

**Results:**

Participants viewed bottled water to be significantly safer to consume than tap water (*p* < 0.001). On a Likert scale from 1 (low risk) to 5 (high risk), “drinking tap water in Nogales, Arizona” received an average score of 4.7, which was significantly higher than the average perceived risk of drinking San Francisco, California tap water (µ = 3.4, *p* < 0.001), and as risky as drinking and driving (µ = 4.8, *p* = 1.00) and drinking Nogales, Sonora, Mexico tap water (µ = 4.8, *p* = 1.00). Ninety-eight percent of participants feared that drinking local tap water could result in illness, 79% did not drink their water because of fear of microbial and chemical contamination and 73% would drink their water if they knew it was safe regardless of taste.

**Conclusions:**

These results suggest that fear of illness from tap-water consumption is an important contributing factor to increased bottled water use. Future efforts should focus on the development of educational and outreach efforts to assess the safety and risks associated with tap-water consumption.

**Supplementary Information:**

The online version contains supplementary material available at 10.1186/s12889-022-14109-5.

## Background

### Preferences for tap water in the U.S.

Bottled water consumption is ubiquitous in the U.S., where 85 million bottles of water are used daily [1]. Several drinking-water studies have shown that an increasing proportion of the U.S. population is choosing an alternative to drinking tap water [1–3], in part due to perceived health risks. In low-income families, an increased use of bottled water over tap water for both drinking and cooking has also been reported [2–5]. Despite prevalent beliefs that consumption of tap water is riskier than bottled water, this does not always match reality. In Nogales, Arizona, where bottled water has traditionally been preferred over drinking water [6], the 2020 consumer confidence report of municipal water indicated that there were no violations for regulated contaminants [7], 7 years after the timeframe of this study (Spring 2012). In 2012, Arizona had 821 systems out of 1,542 with “significant violations” [8] (individualized report for Nogales, AZ not available, to our knowledge). It should be noted that lack of violations does not necessarily indicate lack of health risk for consumers. In the past, major US waterborne outbreaks have been in systems that did not violate regulatory standards [9]. However, the number of violations for some contaminants has changed in more recent years due to stricter regulations or new regulations where there previously were none [10].

### Factors affecting drinking water preferences

There is a myriad of factors that can influence drinking water preferences, including awareness of contamination events, public trust, bottled water branding, convenience, organoleptic preferences (taste, color, smell), and population demographics [11–15]. The public’s concern over the safety of drinking water has grown over the past thirty years due to increased awareness of environmental pollutants, sporadic episodes of waterborne disease outbreaks, and ground water contamination [4, 16–18]. This has led to diminished trust in public water utilities. This can be especially true in rural communities, like Nogales, Arizona, where municipalities have greater challenges in maintaining or achieving adequate water quality [19].

Trust, an antecedent of perceived risk, can influence the acceptability of hazards, with evidence [20] that diminished trust in tap water purveyors due to contamination events or violation of federal drinking water standards [18] may influence perceived risks about municipal drinking water sources. This can ultimately drive drinking water preferences and self-protective behaviors, such as purchasing bottled water or use of pitcher or faucet water filters or the use of point-of-entry (POE) or point-of-use (POU) drinking-water purification systems (which is steadily increasing in use in the U.S.) [21, 22].

Demographics characteristics such as socioeconomic status (SES) [5, 23], gender [4, 20, 24, 25], age [17, 26, 27] and ethnicity [28] may be important factors in understanding risk perception [5, 29]. For example, lack of education or lower household income can place communities in more vulnerable situations in which they endure risks that are greater than other communities, resulting in public health disparities and influencing risk perceptions [23]. Substantial evidence suggests that ethnic minorities (Latino and Pacific Islander) are more concerned than whites about the presence of contaminants in municipal drinking water and have higher consumption rates of bottled or alternative sources of drinking water [24, 30, 31]. In particular, Latinos are more likely than non-Latinos to primarily drink and give their children bottled water because of fear of tap water contamination [3]. However, there is inconsistency in differences between male and female risk perceptions of drinking water source [4, 20, 24, 25].

### Drinking water practices in Nogales, Arizona

Issues of drinking water safety and risk perception are especially important in small, rural communities, such as in Nogales, Arizona, a city with a population of approximately 19,770 on the U.S. and Mexico international border, where 94.8% of the population is Latino [32] and approximately 34% of the participants lived below the federal poverty level in 2010. In the late 1990s, there was concern regarding potential increased prevalence of multiple myeloma and systemic lupus erythematosus, and many believed this to be associated with water contamination [33]. However, since that time very limited research or follow-up has been conducted in that community [34]. In a previous cross-sectional study, we demonstrated that approximately 85% of low-income Latino participants in Nogales, Arizona drank bottled water, and 50% cooked with it [35]. Use of bottled water for activities such as cooking in addition to drinking water in this region may be cultural, in which Mexican immigrants comprise a large proportion of the Nogales Border community. Mexico has the highest rates of bottled water consumption worldwide [36], where the use of bottled water for drinking cooking, and household activities (i.e., showering, washing dishes, brushing teeth) is common.

The preference of bottled over tap water can come at a large economic cost, where the average price of bottled water can be hundreds to thousands of times more expensive than tap water [37, 38]. It may also come at a health cost, where increased bottled water usage may translate to decreased fluoride intake, increasing risk of dental cavities [39]. In the previous study, all participants were WIC recipients indicating they had a low household income, yet used much costlier bottled water for drinking and cooking.

Understanding risk perceptions surrounding drinking water sources for this community has important implications for future outreach efforts and protection of public health. While previous work elucidated higher consumption rates of bottled water than tap water among Latinos in Nogales, Arizona, the risk perceptions driving this preference and how perceptions of these risks compare to those of other everyday activities is unknown. However, we hypothesize that trust in tap water has been eroded in past years due to solvent plumes in the groundwater [40], prior drinking-water violations by water utilities in the region [41, 42], potential contamination from *maquiladoras* (factories that are generally duty- and or tariff-free) just across the U.S.-Mexico Border [43], and increased incidence of certain diseases (i.e., multiple myeloma, lupus) [34]. Data are needed to evaluate these hypotheses and to characterize the decision making behind bottled water preferences and to inform the development of successful public health interventions. Although this study is focused on one historically marginalized community, centering our research on understanding risk perceptions related to preference for drinking bottled water in this target population will help inform design of public health interventions not only for this community but for the numerous other historically marginalized communities with historical mistrust of tap water [44].

### Study objectives

The primary objective was to characterize the risk perceptions of municipal tap water and bottled water and to compare drinking water risk perceptions to those of other activities among low-income families living in Nogales, Arizona.

## Methods

### Study design and population

All study procedures were approved by the University of Arizona Human Subject Protection Program. Between Feb-May 2012, participants were recruited during regular business hours from Mariposa Community Health Center (MCHC) waiting rooms, a chain discount store, and participant referrals. To better understand why low-income families with access to municipal public tap water chose to use their limited resources to drink primarily bottled water, we had the following inclusion criteria: annual household income < $30,000, connected to a public water utility, primarily drank bottled water, and had at least one child living in the home. Individuals whose households were supplied mainly by water from a private well were excluded. MCHC *promotoras* (community health workers) assisted with recruiting families and collecting data. Participants were compensated with $20.

### Questionnaire

A questionnaire was simultaneously developed in Spanish and English by the principal investigator, who is bilingual (P.I. Beamer) and the study team. The questionnaire development was informed by several risk perception studies [4, 45, 46] and administered orally in Spanish (75%) or English (25%), according to the participants’ preference, taking approximately 20–30 min to complete, where participants were eligible to only take the questionnaire once. Three surveyors collected the data. Answers were written down by surveyors as participants expressed their answers. These hard copy surveys were then translated to a digital spreadsheet.

Many of the questions regarding tap and bottled water were developed based on questions from a previous study [5]. However, the investigators added additional statements based upon the criteria for drinking water quality [47], concerns expressed about tap water among community partners, and to better directly compare perceptions between tap and bottled water. Participants were asked to assess their perceptions of risk for different drinking water sources and were asked open-ended questions about possible health outcomes from drinking local tap water. On a Likert scale of 1 (not likely) to 5 (very likely), they were then asked about the likelihood of each health outcome they listed. Participants were also asked to assess levels of dread, a common risk perception measure [46], for these same health outcomes using a Likert scale, 1 (no dread) to 5 (highly dread). Each health outcome was recorded, and five coders classified health outcomes into nine groups based on the responses: gastrointestinal (GI) illness, lupus, parasites/microbes, cancer, chemicals, general infections, allergic reactions, dental problems, and “other.”

Another Likert scale system [46] was used to assess perceived risk and voluntariness of drinking tap water in Nogales, Arizona in comparison with other activities that present a risk (e.g., drinking and driving, smoking, and other activities used in previous risk perception studies [45]) and drinking water in other geographic locations that varied by country, size of city, and distance from Nogales, AZ. These geographical locations included San Francisco, California; Vancouver, British Columbia, Canada; Nogales, Sonora, Mexico; and Guadalajara, Jalisco, Mexico. Participants were asked to assess each activity on a scale of 1 (no risk) to 5 (high risk). A similar scale was used to assess levels of voluntariness (1 = not willing, 5 = willing). The scale was used to elucidate how participants compared risk of consumption of local tap water to other risks. Demographic details (i.e., sex, age, household income, education level, years lived in the U.S., and immigration status) were also collected to examine if any of these variables were associated with participants’ perceived risks and anticipated health outcomes related to drinking tap water in Nogales, Arizona. Using open-ended questions, we assessed the cost of bottled water relative to tap water and identified factors that led to the behavior of using bottled water for drinking and cooking.

### Statistical analysis

All questionnaire responses were hand-coded into STATA® (version 12.1, College Station, TX), which was used for all statistical analyses. Unpaired t-tests were used: 1) determine if statistically significant (α = 0.05) differences occurred in perceived risk by drinking-water source, and 2) determine whether there were statistically significant (α = 0.05) differences among different demographic groups in their level of (dis)agreement with statements regarding tap or bottled water safety. Negative health outcomes that participants anticipated from drinking local tap water were tallied. Means and standard deviations of the perceived likelihood of health outcomes and associated dread were then calculated.

To assess significant differences (α = 0.05) in perceived risks and voluntariness to engage in a number of activities, including drinking local tap water and drinking water in different geographic regions, a one-way analysis of variance (ANOVA) followed by Scheffe’s post-hoc test with adjustment for the number of pairwise comparisons was used. These analyses were conducted by stratifying by demographic characteristics to assess whether the relationships described above were influenced by demographic variables.

## Results

### Demographics

All participants (*n* = 90) answered all questions on perceived risks associated with drinking-water sources and health outcomes. A summary of participants’ distribution by demographic characteristics is provided in Table 1. All study participants were Latino, similar to the proportion of Latino individuals in the city (94.8%) [32]. A majority were women (83%, 24/29), and 53% had an annual household income of less than $15,000, slightly lower than the medium household income in 2020 ($29,043) [32].

**Table 1:** Demographic characteristics of study population (*n* = 90)

Characteristics	n	%
**Gender**		
Female	75	83
Male	15	17
**Age**		
< 35 years	44	49
≥ 35 years	46	51
**Immigration Status**		
Immigrant (born outside U.S.)	44	49
Non-Immigrant (born in U.S.)	46	51
**Annual household income**		
< $15,000	48	53
Between $30,000 and $15,000^a^	42	47
**Education level**		
≤ 8th grade	43	48
9-12th grade	27	30
Some college	20	22
**Years lived in the U.S**		
0–10 yrs	22	24
11–20 yrs	35	39
> 20 yrs	33	37

^a^An inclusion criterion was that participants must have a household income of $30,000 or less, explaining why no participants had a reported household income greater than $30,000

The sample population had a mean age of 39 (SD = 17) years, with a majority of participants reporting education levels at or below 8^th^ grade and only 22% (20/90) having attended college. Among single-parent families (32%, 29/90), several were headed by females (83%, 24/29) and a few by males (17%, 5/29). Immigration statuses of participants were almost evenly distributed with 49% immigrants (born outside the U.S.) and 51% non-immigrants (born in the U.S.), where the majority of immigrants (72%, 32/90) had been living in the U.S. for more than 10 years.

### Sources of water-related risk perceptions

Using a Likert scale from 1 (strongly disagree) to 5 (strongly agree), participants viewed bottled water and other purchased sources of drinking water to be significantly safer to drink than local tap water (t = 3.7, df = 29, *p* < 0.001) (Table 2), and participants generally strongly disagreed with the statement, “It is safe to drink my tap water,” and strongly agreed with the statement, “It is safe to drink bottled water” (Fig. S1A). Participants generally strongly agreed that friends or family told them not to drink local tap water (Table 2; Fig. S1B, additional file 1). Fifty-four percent (49/90) reported drinking tap water when they were younger but having changed to bottled water. Distributions of responses for all water-related risk perception questions can be seen in Fig. S2 (additional file 1).

**Table 2:** Summary of participants’ perception scores (*n* = 90) regarding different water sources on a Likert scale from 1 (strongly disagree) to 5 (strongly agree)

Activity	Mean	SD
***Tap water perceptions***		
I’m happy with the taste of my tap water	2.0	1.4
I’m happy with the odor of my tap water	3.0	1.6
I’m happy with the color of my tap water	3.1	1.5
I’m happy with the clarity of my tap water	3.2	1.6
I drink my tap water	2.2	1.6
It is safe to drink my tap water	2.2	1.6
I trust my tap water company to provide me with safe drinking water	2.6	1.7
My tap water has too much chlorine	3.4	1.5
My tap water is too hard	3.5	1.6
Friends or family have told me not to drink tap water	4.5	1.1
***Bottled water perceptions***		
It is safe to drink water vended at water stations or at the store	3.5	1.5
It is safe to drink bottled water	3.9	1.4
The way I store my water keeps my water clean	4.3	1.3
I use bottled water or other sources of water (not tap) for drinking	4.4	1.3
The water containers I use are clean	4.5	1.0

No significant differences in perceived risks of drinking-water sources by sex, age, immigration status, annual household income, education level, and number of years participant had been living in the U.S. (data not shown). Furthermore, similar results were observed when analyses were stratified by sex, age, immigration status, annual household income, education level, and number of years the participant had been living in the U.S. (data not shown).

### Rationale for drinking water choice

Most of the participants (79%, *n* = 71) stated that their primary reason for not drinking local tap water was fear of chemical or microbial contamination that may result in illness, compared to only 17% (*n* = 15) who preferred the taste of bottled water, consistent with a generally strong disagreement with the statement, “I’m happy with the taste of my tap water” (Fig. S1B). However, 73% (*n* = 66) stated that they would drink their tap water if they knew it was not contaminated, even if they did not like the taste.

### Likelihood and dread of health outcomes anticipated by participants

The majority of participants (98%) believed that drinking local tap water could result in adverse health outcomes, including GI illness, lupus, or cancer, where GI illness and lupus were the two most frequently listed outcomes (Table 3). While chemicals were not listed as one of the main outcomes of concern, those who listed this concern had an average dread score of 4.3 (1 = no dread, 5 = highly dreaded), while the average scores for GI illness and lupus were 3.6 and 4.2, respectively (Table 3).

**Table 3:** Summary of participants’ perceived possible health effects from drinking tap water in Nogales, Arizona, and their perceptions of the likelihood and their dread of each health outcome on a Likert scale from 1 (not likely/no dread) to 5 (very likely/highly dread).^a^

			**Perceived Likelihood**		**Dread**	
**Outcome**	**n**	**%**	**Mean**	**SD**	**Mean**	**SD**
GI illnesses	74	45	3.7	1.1	3.6	1.0
Lupus	21	13	4.1	1.0	4.2	0.8
Parasites/Microbes	16	10	2.8	1.1	2.7	1.0
Cancer	15	9	3.8	0.9	3.6	1.1
Other	10	6	3.3	1.2	3.4	0.7
Chemicals	9	5	4.3	0.9	4.3	0.9
General infections	8	5	2.8	1.0	2.9	1.0
Allergic reactions	8	5	2.4	1.3	2.4	0.9
Dental problems	5	3	3.4	1.8	3.4	1.8

^a^Participants could list more than one health outcome, explaining a total *n* > 90

### Risk and voluntariness comparison of local tap water consumption to other activities

On a Likert scale from 1 (low risk) to 5 (high risk), participants perceived drinking tap water in Nogales, Arizona, to be as risky as drinking tap water in Nogales, Mexico, and significantly less risky than drinking tap water in Vancouver, Canada, or in San Francisco, California (Table 4). Participants also perceived consumption of tap water in Nogales, Arizona to be as risky as drinking and driving and significantly riskier than smoking, riding a motorcycle, playing American football, and listening to loud music (Table 4). While participants considered drinking local tap water as risky as exposure to pesticides, they considered it riskier than using Raid™ (Table 4). Participants’ voluntariness for firing a gun or listening to loud music was significantly greater than for drinking tap water in Nogales, Arizona (Table 5).

**Table 4:** Summary of perceived risk scores (*n* = 90) of drinking tap water in Nogales, Arizona relative to several other voluntary activities on a Likert scale from 1 (low risk) to 5 (high risk)

Activity	Mean	SD	Mean difference	*P*-value
***Drinking-water activities***				
Drinking tap water in Nogales, AZ, USA	4.7	0.8	Ref	Ref
Drinking tap water in Guadalajara, Jalisco, México	4.1	1.1	0.6	0.431
Drinking tap water in Nogales, Sonora, México	4.8	0.6	-0.1	0.972
Drinking tap water in San Francisco, CA, USA	3.4	1.4	1.3	< 0.001*
Drinking tap water in Vancouver, British Columbia, Canada	3.4	1.3	1.3	< 0.001*
Drinking water from a well	4.6	1.0	0.1	1.00
***Other activities***				
Drinking and driving	4.8	0.6	-0.2	1.00
Driving a car	3.1	1.3	1.6	< 0.001*
Exposure to pesticides	4.7	0.6	0.0	0.984
Firing a gun	4.3	0.9	0.4	0.972
Listening to loud music	3.8	1.4	0.8	0.020*
Playing American football	3.8	1.1	0.9	0.022*
Riding a motorcycle	2.6	1.3	2.1	< 0.001*
Riding in a car without a seatbelt	4.4	0.9	0.3	0.971
Smoking	3.5	1.2	1.2	< 0.001*
Using Raid™ (insecticide)	3.3	1.3	1.4	< 0.001*

^*^Statistically significant (α = 0.05)

**Table 5:** Summary participants’ voluntariness (*n* = 90) of drinking tap water in Nogales, Arizona relative to several other activities from a Likert scale from 1 (low willing) to 5 (willing)

Activity	Mean	SD	Mean difference	F statistic	*P*-value
***Drinking-water activities***					
Drinking tap water in Nogales, AZ, USA	1.7	1.2	Ref		Ref
Drinking tap water in Guadalajara, Jalisco, México	1.9	1.3	-0.2	0.5	0.982
Drinking tap water in Nogales, Sonora, México	1.9	1.4	-0.2	0.5	0.972
Drinking tap water in San Francisco, CA, USA	2.5	1.4	-0.8	1.3	0.244
Drinking tap water in Vancouver, British Columbia, Canada	2.3	1.3	-0.6	0.7	0.823
Drinking water from a well	2.1	1.4	-0.4	0.6	0.994
***Other activities***					
Drinking and driving	1.1	0.5	-0.6	0.7	0.842
Driving a car	4.5	1.2	-2.8	5.3	< 0.001*
Firing a gun	2.8	1.6	-1.1	5.1	0.002*
Listening to loud music	2.7	1.5	-1.1	4.2	0.010*
Playing American football	2.3	1.4	-0.6	0.6	0.922
Riding in a car without a seatbelt	1.4	0.9	-0.3	0.5	0.971
Smoking	1.4	1.0	-0.3	0.5	0.983
Using Raid™ (insecticide)	2.4	1.3	-0.8	1.2	0.312

^*^ Statistically significant (α = 0.05)

### Self-protective behaviors

As a self-protective measure, all participants reported purchasing bottled water, and 18% (*n* = 16) of households used it for cooking as well. On average, each household reported spending $96 per 100 gallons of bottled water annually, more than two hundred times the cost of tap water ($0.45 per 100 gallons). The majority of participants (77%, 69/90) reported using faucet filters prior to drinking or cooking. Those who were supplied with water from a purveyor with a previous record of safety violations were more likely to boil their tap water before using it for drinking or cooking (8%, 7/90). None of the participants boiled or filtered bottled or other purchased water before using it.

## Discussion

In this study, participants had negative perceptions about the safety of local tap water (Tables 2 and 3, Fig. S1), believing it could result in both acute and chronic diseases (i.e., GI illness, lupus, and cancer) (Table 3), and perceived bottled and other sources of purchased water to be safer for consumption. In fact, 73% (*n* = 66) stated that they would drink their tap water if they knew it was not contaminated, even if they did not like the taste. The perception of bottled and other purchased water to be safer for consumption may be attributed to significantly lower water-related outbreaks in bottled water than in tap water sold in U.S. [48] and the fact that bottled water purveyors are more likely to promote their products in regions similar to the Nogales, Arizona area as a safer alternative to tap water [49].

### Risk perceptions of drinking water by geographical location

Participants’ perceptions of tap water as being unsafe did not widely apply to all geographical locations (San Francisco, CA; Vancouver, British Columbia, Canada; Nogales, Sonora, México; Guadalajara, Jalisco, México). Participants perceived the risks of consuming tap water to be lower in other places in the U.S. (San Francisco, CA) and in Vancouver, British Columbia, Canada, but much higher in Nogales, Mexico and Guadalajara, Jalisco, Mexico (Table 4).

In comparison to drinking Nogales, Arizona tap water, participants considered drinking tap water in Nogales, Mexico (with a history of experiencing recurring water quality issues) to be as risky (Table 4). Mexican immigrants comprise a large proportion of the population living in Nogales, Arizona, and previous studies have demonstrated decreased tap water use among immigrants due to fear of subsequent illness [3]. It should be noted in this study that there was no statistically significant difference by immigration status. However, the consistency in risk perceptions for tap water in Nogales, Arizona and Nogales, Mexico provides evidence of cross-border perceptions of municipal water quality and safety in this region [42]. The perception of tap water to be unsafe extends beyond Nogales, Arizona and has been reflected in surveys of other U.S. communities, where greater proportions of Hispanic (16.39%, 876/5,048) and Black participants (8.48%, 434/5,132) viewed tap water as unsafe than non-Hispanic White participants (5.07%, 1,338/26,407) [50]. However, it should be noted that the proportion of Hispanic participants (16.39%) who perceived tap water to be unsafe in the larger U.S. study conducted by Javidi & Pierece (2018) is notably smaller than the proportion of participants in this study who stated that their primary reason for not drinking local tap water was fear of chemical or microbial contamination that may result in illness (79%, *n* = 71). This reiterates that drinking water perceptions are not homogeneous across communities or ethnic and racial groups, demonstrating the value in studying the risk perceptions of specific low-income, marginalized communities with historic water quality challenges.

The fear of tap water in Nogales, Arizona seemed to be shared by the community at large, where many participants reported being told by friends and family not to drink their tap water (Fig. S1, Table 2). Nogales, Arizona has had drinking-water wells closures due to chemical contamination [41] and there are concerns that increases in number of *maquiladoras* across the Border in Sonora may lead to contaminated groundwater through improper disposal of solvents used by these factories [43]. In addition, several small water purveyors in the area had reported prior drinking-water violations for noncompliance [51]. As a result, many bottled water purveyors in Nogales, Arizona have promoted their product to be a better and safer alternative to local tap water. Many participants reported drinking tap water when they were younger and later changing to bottled water, possibly due to these contamination events personal experiences, new beliefs, or via the influence of advertising. Individuals tend to adopt the attitudes or behaviors of others in the same social networks or communities [18, 52, 53].

### Perceptions of negative health outcomes from drinking tap water

Most participants feared that drinking their tap water could result in adverse health effects, like GI illnesses, cancer, and lupus, where lupus was the most frequently listed potential health outcome and with one of the highest average dread scores (Table 3). While there are several studies that report associations between contaminated drinking water and GI illnesses [16, 54] and some types of cancers (liver, lung, bladder and kidney) [55], there is limited evidence showing or describing the association between contamination of drinking water and lupus. Drinking water was identified as a statistically significant risk factor for systemic lupus erythematosus (SLE) in a study in the Anhui Province, China, where those who drank pond or well water had 2.04 (1.18–3.51) greater odds of SLE, relative to those who drank running water [56]. However, a mechanistic hypothesis was not offered.

An animal model has been used to demonstrate the influence of drinking water pH on gut microbiota, with subsequent effects on progression of lupus in mice [57]. Other potential risk factors for SLE development include exposure to solvents, trichloroethylene (TCE), and pesticides, but with less evidence than other risk factors (i.e., smoking and crystalline silica exposure) [58]. Nogales, Arizona has a TCE plume [40], and TCE has been detected in the breast milk of mothers in Nogales, AZ and in household water [35]. There is also evidence of transgenerational effects of environmental exposures [59], where epigenetic changes resulting from exposures can pose increased lupus risk for multiple generations. While the causal relationship between chemical contamination or characteristics of local tap water and the prevalence of lupus has yet to be elucidated, the Nogales area experiences rates of lupus that are 4 to 7 times above the national average [34]. Further investigation is needed to explore whether water sources contribute to this health burden.

### Risk perceptions of Nogales, AZ tap water consumption compared to other activities

In comparison to other activities, participants perceived drinking local tap water to be as risky as activities with risk of injury or death. For example, participants viewed local tap-water consumption to be as risky as drinking and driving, a high-risk activity [60]. Participants also perceived drinking local tap water to be more risky than using Raid™ and smoking, both of which pose chronic adverse health outcomes [61, 62]. Interestingly, these two activities perceived as less risky than drinking tap water are risk factors for lupus development [58], a top health concern in this community within the context of water consumption.

### Limitations

The lack of statistically significant differences in risk perception of tap water by demographic characteristics could be due in part to a relatively small sample size (*n* = 90), and even smaller subsets of participants for comparing across demographics. For example, most of the households were of families with both parents (68%, 61/90), where the men were more likely to be at work and unavailable to participate or opted not to take part in the study. This made it challenging to compare risk perceptions between male and female participants. The low participation of males in population surveys, especially from ethnic or racial minorities, has been observed in other studies [3, 28]. While analyses were stratified by sex due to evidence that women may tend to experience higher risk levels than perceived men [23, 46], the low number of male participants may have contributed to a lack of any statistically significant finding.

The small sample size in this case also challenged the use of other statistical approaches, such as the use of linear regression models, where we would have been limited in the number of explanatory variables and many of them are highly correlated. Future studies with larger sample sizes should consider the use of other methods for comparing the magnitude and direction of relationships between demographic variables and risk perceptions for informing public health outreach efforts. However, it should be noted that while the sample size is small, the participants of this study were from a low-income community in a city on the U.S.-Mexico Border, a community that is difficult to access without the assistance of and collaboration with trusted community members (i.e., promotoras). Considering the challenges in forming these community connections and the limited data available on low-income Border communities, these data hold important public health value.

It should also be noted that our study population is not intended to represent the city of Nogales; rather, the objective was to capture risk perceptions of low-income community members who primarily drank bottled water in order to develop future interventions. It was therefore challenging to evaluate whether our specific study participants were representative of this specific community within Nogales. In comparison to the city, our recruited population did indeed have lower income than the city: more than half (53%) of the participants had a household income of < $15,000, lower than the median household income reported in 2020 ($29,043). This indicates that our participants may experience a greater economic tradeoff, relative to the rest of the city, of accepting the larger cost of drinking bottled water relative to tap water in response to perceived risks from drinking tap water.

In addition to small sample size, another possible explanation for a lack of statistically significant risk perception differences by demographic characteristics is that perceived risks are homogeneous in the study population due to the inclusion criteria. This hypothesis is supported by an argument made by Greenberg and Schneider showing that communities “stressed” by environmental risks tend to have relatively homogenous risk perceptions [63]. Additionally, the participants in this study were of low-income and reported primarily drinking bottled water. If the risk perceptions among the participants are truly homogeneous, it does not mean risk perceptions are homogeneous in other communities or among those in this community that do drink the tap water. The purpose of the limited inclusion criteria was to better understand why low-income families in a historically marginalized community use their limited resources to purchase bottled water and what information would need to be provided to increase their trust in municipal tap water. More data are needed to investigate how risk perceptions among the Nogales, Arizona Latino community may differ from other communities who primarily consume bottled water to inform community-specific interventions or educational programs that may be more effective. It should also be noted that the survey utilized in English and Spanish was not formally evaluated for bias or differences in interpretation between the two versions. Future work is needed to evaluate and validate survey instruments for reliably capturing risk perceptions in communities where multiple languages are spoken.

## Conclusions

Participants generally perceived tap water in Nogales, Arizona to be unsafe for drinking compared to bottled water and did not associate drinking bottled water with any perceived health outcome. Fear of illness from tap water contamination is an important contributing factor to increased use of bottled water in this community and may possibly be driven by social behaviors within community networks in the Nogales region. While tap water is perceived as higher risk than bottled water, this may not be true in every case. More training programs can be implemented to inform community members about water quality in Nogales, Arizona. Such programs can partner with and utilize *promotoras* to educate low-income families on water quality monitoring and regulatory standards and the lower cost of local tap water.

More research is needed to assess tap water consumption risk perceptions in other U.S.- Mexico Border communities and in other historically marginalized communities to increase participation to investigate risk perception differences among demographic groups, such as males vs. females. Understanding the influences on risk perceptions of tap water and the values that communities place on water quality and health will inform more effective strategies for improving and protecting public health and potentially allow families with limited resources to utilize more of their resources on healthier food or health care.

## Supplementary Information

**Additional file 1: Figure S1.** Comparison of agreement with A) safety of tap vs. bottled water, and B) statements regarding advice from family and friends about drinking tap water and perception of taste of tap water.** Figure S2.** Distribution of responses to perception questions.

**Additional file 2.** Data file for questionnaire responses

**Additional file 3.** Drinking Water Risk Perception Questionnaire.

## Data Availability

The data analyzed during this study are included in this published article and its supplementary information files.
