# Supplementary material for: Risk perceptions of drinking bottled vs. tap water in a low-income community on the US-Mexico Border
Source: BMC Public Health. 2022 Sep 9;22:1712. doi: 10.1186/s12889-022-14109-5 (PMC9463786; doi:10.1186/s12889-022-14109-5)
Supplement: Supplementary file 1 — Additional file 1: Figure S1. Comparison of agreement with A) safety of tap vs. bottled water, and B) statements regarding advice from family and friends about drinking tap water and perception of taste of tap water. Figure S2. Distribution of responses to perception questions. [file 12889_2022_14109_MOESM1_ESM.docx]

| **A**  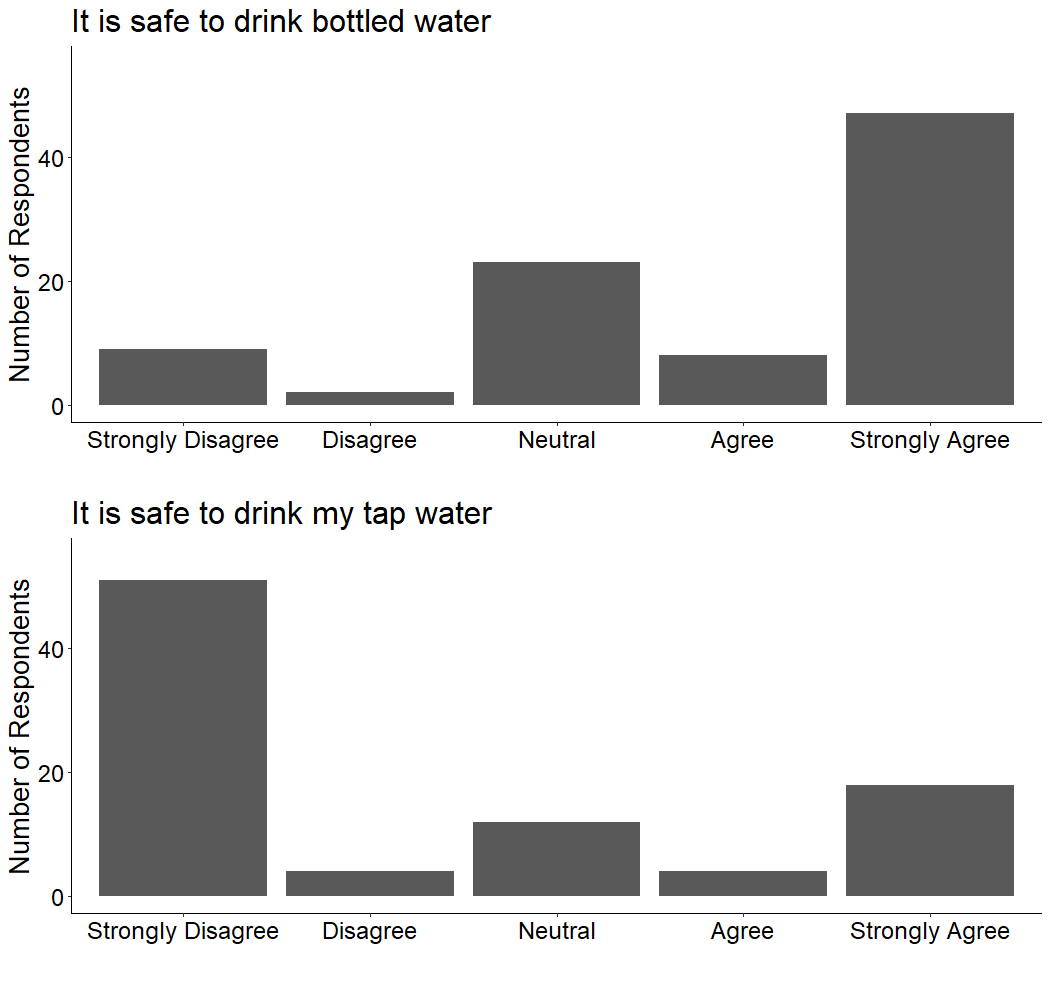 |
| --- |
| **B**  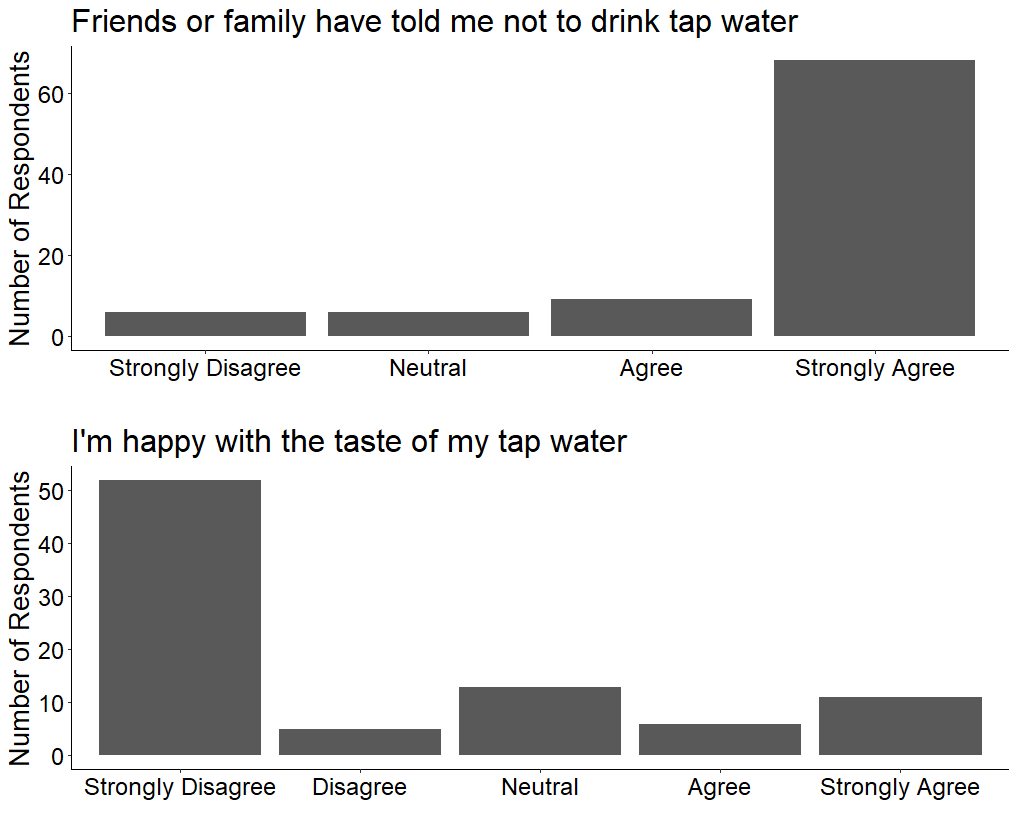 |

**Figure S1.** Comparison of agreement with A) safety of tap vs. drinking water, and B) statements regarding advice from family and friends about drinking tap water and perception of taste of tap water


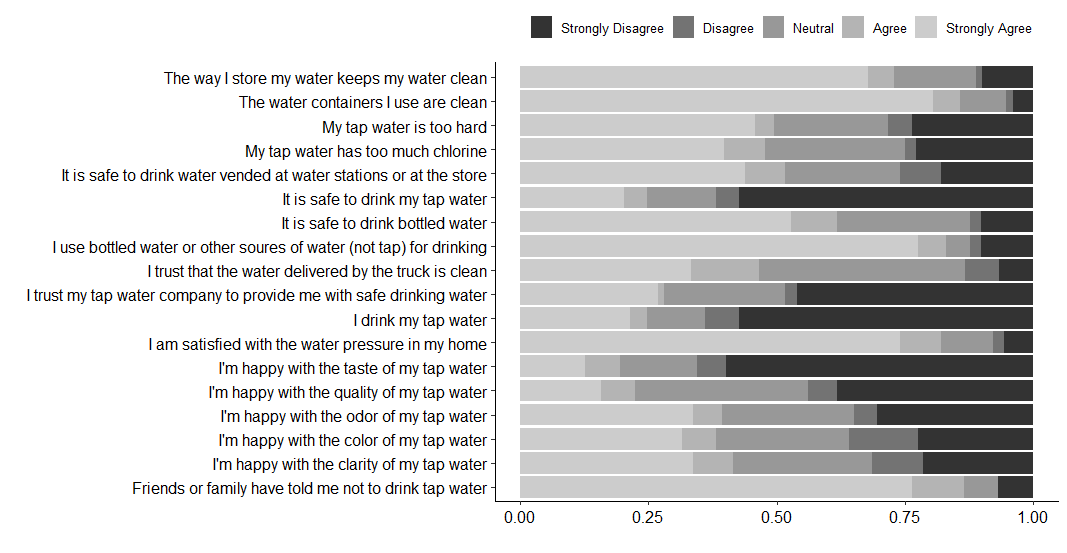


**Figure S2.** Distribution of responses to perception questions
