# Supplementary material for: Risk perceptions of drinking bottled vs. tap water in a low-income community on the US-Mexico Border
Source: BMC Public Health. 2022 Sep 9;22:1712. doi: 10.1186/s12889-022-14109-5 (PMC9463786; doi:10.1186/s12889-022-14109-5)
Supplement: Supplementary file 3 — Additional file 3. Drinking Water Risk Perception Questionnaire. [file 12889_2022_14109_MOESM3_ESM.docx]

**Drinking Water Study in Nogales, AZ**

**Spring 2012**

Thank you for taking time to meet with me today. I will ask questions about your water drinking habits, reasons for choosing alternative water sources for drinking and cooking, and potential risks associated with different water sources. Please feel free to ask me if my questions do not make sense or you need more information. You are free to skip any questions you do not feel comfortable answering.

*********************************************************************************

**INTERVIEWER FILL OUT:**

PARTICIPANT ID:

NAME OF INTERVIEWER:______________________________

DATE OF QUESTIONAIRE ADMINISTRATION: ______________________

TIME QUESTIONNAIRE BEGAN: _____________

TIME QUESTIONNAIRE ENDED: _____________

INTERVIEW IN ENGLISH? Yes ___ No ___

*********************************************************************************

**PART I*.***

***This section pertains to how you perceive risk. Risk is an evaluation of both a hazard to health and the likelihood that it will occur. I am going to give you a list of activities and I want you to tell me how risky you feel they are. I am not asking what you think OTHER people feel about the activities, but rather what YOU think about them in your life. Please note that you do not have to actually have participated in any of the activities in order to give your opinion on them.***

1. Please rate the following activities on a 1-5 scale in terms of how risky you feel it is.

1= Low Risk, 3= Moderate Risk, and 5= High Risk.

| **Activity** | **Low Risk** |  | **Moderate Risk** |  | **High Risk** |
| --- | --- | --- | --- | --- | --- |
| **1a.** Riding a motorcycle | 1 | 2 | 3 | 4 | 5 |
| **1b.** Driving a car | 1 | 2 | 3 | 4 | 5 |
| **1c.** Playing American football | 1 | 2 | 3 | 4 | 5 |
| **1d**. Firing a gun | 1 | 2 | 3 | 4 | 5 |
| **1e.** Using Raid™ (insecticide) | 1 | 2 | 3 | 4 | 5 |
| **1f.** Listening to loud music | 1 | 2 | 3 | 4 | 5 |
| **1g.** Drinking water from a well | 1 | 2 | 3 | 4 | 5 |
| **1h.** Riding in a car without a seatbelt | 1 | 2 | 3 | 4 | 5 |
| **1i.** Smoking | 1 | 2 | 3 | 4 | 5 |
| **1j.** Drinking and driving | 1 | 2 | 3 | 4 | 5 |
| **1k.** Exposure to pesticides | 1 | 2 | 3 | 4 | 5 |
| **1l.** Drinking tap water in Vancouver, British Columbia, Canada | 1 | 2 | 3 | 4 | 5 |
| **1m.** Drinking tap water in Guadalajara, Jalisco, México | 1 | 2 | 3 | 4 | 5 |
| **1n.** Drinking tap water in Nogales, Sonora, México | 1 | 2 | 3 | 4 | 5 |
| **1o.** Drinking tap water in Nogales, AZ, USA | 1 | 2 | 3 | 4 | 5 |
| **1p.** Drinking tap water in San Francisco, CA, USA | 1 | 2 | 3 | 4 | 5 |

***Now I would like you to consider how likely you are to voluntarily perform each of the activities listed below. Please base your answers on whether you would perform them now, not whether you have done it in the past.***

2. Please rate the following activities again on a 1-5 scale. 1= Not Voluntarily (Not Willing), 3= Neutral, and 5= Voluntarily (Willing).

| **Activity** | **Not Voluntarily (Not Willing)** |  | **Neutral** |  | **Voluntary (Willing)** |
| --- | --- | --- | --- | --- | --- |
| **2a.** Riding a motorcycle | 1 | 2 | 3 | 4 | 5 |
| **2b.** Driving a car | 1 | 2 | 3 | 4 | 5 |
| **2c.** Playing American football | 1 | 2 | 3 | 4 | 5 |
| **2d**. Firing a gun | 1 | 2 | 3 | 4 | 5 |
| **2e.** Using Raid (insecticide) | 1 | 2 | 3 | 4 | 5 |
| **2f.** Listening to loud music | 1 | 2 | 3 | 4 | 5 |
| **2g.** Drinking water from a well | 1 | 2 | 3 | 4 | 5 |
| **2h.** Riding in a car without a seatbelt | 1 | 2 | 3 | 4 | 5 |
| **2i.** Smoking | 1 | 2 | 3 | 4 | 5 |
| **2j.** Drinking and driving | 1 | 2 | 3 | 4 | 5 |
| **2k.** Exposure to pesticides | 1 | 2 | 3 | 4 | 5 |
| **2l.** Drinking tap water in Vancouver, British Columbia, Canada | 1 | 2 | 3 | 4 | 5 |
| **2m.** Drinking tap water in Guadalajara, Jalisco, México | 1 | 2 | 3 | 4 | 5 |
| **2n.** Drinking tap water in Nogales, Sonora, México | 1 | 2 | 3 | 4 | 5 |
| **2o.** Drinking tap water in Nogales, AZ, USA | 1 | 2 | 3 | 4 | 5 |
| **2p.** Drinking tap water in San Francisco, CA, USA | 1 | 2 | 3 | 4 | 5 |

**PART II.**

***The next questions will help us evaluate your level of comfort with the health effects that you associate with drinking tap water.***

3. In what way(s) can drinking your tap water be harmful to your health?

**[Note to Interviewer: Please write down each health effect mentioned to you]**

| **1.** |
| --- |
| **2.** |
| **3.** |
| **4.** |
| **5.** |

***Now I would like you to consider how likely you think that each health effect you mentioned can occur.***

4. Please rate the health effects on a 1-5 scale: 1= Not likely, 3= Likely, 5= Very likely.

**[Note to Interviewer: Please write down each health effect mentioned above in the table below]**

| **Health Effect** | **Not Likely** |  | **Likely** |  | **Very Likely** |
| --- | --- | --- | --- | --- | --- |
| **1.** | 1 | 2 | 3 | 4 | 5 |
| **2**. | 1 | 2 | 3 | 4 | 5 |
| **3**. | 1 | 2 | 3 | 4 | 5 |
| **4**. | 1 | 2 | 3 | 4 | 5 |
| **5**. | 1 | 2 | 3 | 4 | 5 |

***Now I would like you to consider how much you dread each health effect you mentioned.***

5. Please rate the health effects again on a 1-5 scale. 1= Not concerned/do not dread, 3= Concerned/dread, 5= Highly concerned/highly dread.

**[Note to Interviewer: Please write down each health effect mentioned above in the table below]**

| **Health Effect** | **Not concerned/**  **no dread** |  | **Concerned/**  **dread** |  | **Highly concerned/**  **highly dread** |
| --- | --- | --- | --- | --- | --- |
| **1**. | 1 | 2 | 3 | 4 | 5 |
| **2**. | 1 | 2 | 3 | 4 | 5 |
| **3**. | 1 | 2 | 3 | 4 | 5 |
| **4** | 1 | 2 | 3 | 4 | 5 |
| **5**. | 1 | 2 | 3 | 4 | 5 |

**PART III.**

***Now I would like to ask you a few questions about what you think about different water sources such as tap water and bottled water, and the risks you associate with them.***

6. Please rate the following statements using the following scale in terms of how much you agree or disagree. 1= Strongly Disagree, 3= Neutral, 5= Strongly Agree, 79= Not applicable.

| **Activity** | **Strongly Disagree** |  | **Neutral** |  | **Strongly Agree** | **Not Applicable** |
| --- | --- | --- | --- | --- | --- | --- |
| **6a.** I’m happy with the quality of my tap water | 1 | 2 | 3 | 4 | 5 | 79 |
| **6b.** I’m happy with the *taste* of my tap water | 1 | 2 | 3 | 4 | 5 | 79 |
| **6c.** I’m happy with the *color* of my tap water | 1 | 2 | 3 | 4 | 5 | 79 |
| **6d**. I’m happy with the *odor* of my tap water | 1 | 2 | 3 | 4 | 5 | 79 |
| **6e.** I’m happy with the *clarity* of my tap water | 1 | 2 | 3 | 4 | 5 | 79 |
| **6f.** Friends or family have told me not to drink tap water | 1 | 2 | 3 | 4 | 5 | 79 |
| **6g.** I trust my tap water company to provide me with safe drinking water | 1 | 2 | 3 | 4 | 5 | 79 |
| **6h.** I drink my tap water | 1 | 2 | 3 | 4 | 5 | 79 |
| **6i.** It is safe to drink bottled water | 1 | 2 | 3 | 4 | 5 | 79 |
| **6j.** I use bottled water or other sources of water (not tap) for drinking | 1 | 2 | 3 | 4 | 5 | 79 |
| **6k.** It is safe to drink water vended at water stations or at the store | 1 | 2 | 3 | 4 | 5 | 79 |
| **6l.** I trust that the water delivered by the truck is clean | 1 | 2 | 3 | 4 | 5 | 79 |
| **6m.** The water containers I use are clean | 1 | 2 | 3 | 4 | 5 | 79 |
| **6n.** I am satisfied with the water pressure in my home | 1 | 2 | 3 | 4 | 5 | 79 |
| **6o.** My tap water has too much chlorine | 1 | 2 | 3 | 4 | 5 | 79 |
| **6p.** My tap water is too hard | 1 | 2 | 3 | 4 | 5 | 79 |
| **6q.** It is safe to drink my tap water | 1 | 2 | 3 | 4 | 5 | 79 |
| **6r.** The way I store my water keeps my water clean | 1 | 2 | 3 | 4 | 5 | 79 |

**Open-ended questions**

7. Why do you choose to drink other sources of water, for example bottled water, instead of your tap water?

8. In what way(s) is bottled water or other sources of purchased water “safer to drink” than tap water?

9. What would change your mind about drinking your tap water?

10. What would change your mind about drinking bottled water or from other sources?

**PART IV.**

***This section will ask you about how frequently you experience gastrointestinal illnesses and how you treat them. Examples of gastrointestinal illnesses are diarrhea, nausea, vomiting, abdominal pain, and abdominal cramps.***

**[Note to interviewer: Please read out loud the definition of diarrhea provided below]**

*The National Institutes of Health describes diarrhea as loose, watery stools (not solid). Having diarrhea means passing loose stools three or more times a day.*

11. How frequently do you have bowel movements in a day?

0= None

1= Once

2= Twice

3= Three times

4 = Other _________________

89= Don’t know

99= Refused

12. Has anyone in the household, including you, had episodes of diarrhea in the past week?

0= No 89 = Don’t know

1= Yes 99= Refused

13. How frequently do you have diarrhea?

0= Infrequently (having diarrhea for less than 3 days within 1 week)

1= Frequently (having diarrhea for 3 days within 1 week)

2= Very frequently (having diarrhea for more than 3 days within 1 week)

89 = Don’t know

99= Refused

14. How frequently do your children have diarrhea?

0= Infrequently (having diarrhea for less than 3 days within 1 week)

1= Frequently (having diarrhea for 3 days within 1 week)

2= Very frequently (having diarrhea for more than 3 days within 1 week)

89 = Don’t know

99= Refused

15. The last time you or anyone in your household had diarrhea, what was the cause? Mark all that apply.

0= Nothing

1= Water

2= Food

3= Illness

4= Not sure

5= Other _________________

89 = Don’t know

99= Refused

16. The last time you or anyone in your household had diarrhea, how was it treated? Mark all that apply.

0= Nothing

1= Pepto-Bismol

2= Other over the counter medication _________________

3= Prescription medication

4= Tea

5= Herbal remedies besides tea

6= Juice

7= Water

8= Mineral water

9= Soda

10= Gatorade

11= Other _________________

89= Don’t know

99= Refused

17. How frequently do you have vomiting episodes?

0= Infrequently (less than 2 vomiting episodes a week)

1= Frequently (2 vomiting episodes a week)

2= Very frequently (more than 2 vomiting episodes a week)

89 = Don’t know

99= Refused

18. How frequently do your children have vomiting episodes?

0= Infrequently (less than 2 vomiting episodes a week)

1= Frequently (2 vomiting episodes a week)

2= Very frequently (more than 2 vomiting episodes a week)

89 = Don’t know

99= Refused

19. The last time you or anyone in your household had vomiting, what would you say was the cause? Mark all that apply.

0= Nothing

1= Water

2= Food

3= Illness

4= Not sure

5= Other _________________

89 = Don’t know

99= Refused

20. The last time you or anyone in your household had vomiting, how was it treated? Mark all that apply.

0= Nothing

1= Pepto-Bismol

2= Other over the counter medication _________________

3= Prescription medication

4= Tea

5= Herbal remedies besides tea

6= Juice

7= Water

8= Mineral water

9= Soda

10= Gatorade

11= Other _________________

89= Don’t Know

99= Refused

21. Have you ever visited a doctor for a gastrointestinal illness that you thought was caused by drinking contaminated water?

0= No

1= Yes

89= Don’t know

99= Refused

22. Have you or anyone in your household gotten sick from drinking tap water in Nogales, AZ? **[If answer is “No”, skip to part V]**

0= No

1= Yes

89= Don’t know

99= Refused

23. Where was the tap water from?

1= Own home

2= Restaurant

3= Friend/Family home

4= Work

89= Don’t know

99 = Refused

**PART V.**

***This section will ask you about the water sources you use for drinking and cooking, where you get it from, and how frequently you obtain it.***

24. What company supplies your tap water?

1= Rio Rico Utilities Inc.

2= Arizona American Water Co.

3= Valle Verde Water Co.

4= Nogales Water Department

5= Lakewood Water Co.

6= Donahue's Water Maid

7= Baca Float Water Co. Inc.

8= Other _________________

89= Don’t know

99= Refused

25. How much money do you spend on tap water per month? _________________

**[Note to interviewer: Please help interviewee calculate the amount]**

26. How often do you buy bottled or other types of purchased water?

1= Daily

2= Weekly

3= Bi-weekly

4= Monthly

5= Yearly

89= Don’t know

99= Refused

27. How much does it cost you to buy water per week? _________________

**[Note to interviewer: Please help interviewee calculate the amount]**

28. How frequently do you use the following sources for *cooking*?

**Use the following scale: 0= Never, 1= Rarely, 2= Frequently, 3= Always**

**[Note to interviewer: mark primary source]**

| **Water Source** | **Never** | **Rarely** | **Frequently** | **Always** |
| --- | --- | --- | --- | --- |
| **28a.** Public water system | 0 | 1 | 2 | 3 |
| **28b.** Bottled water from the store | 0 | 1 | 2 | 3 |
| **28c.** Privately owned well | 0 | 1 | 2 | 3 |
| **28d.** Delivered by a company | 0 | 1 | 2 | 3 |
| **28e.** Outdoor faucet or hose | 0 | 1 | 2 | 3 |
| **28f.** Vending machine | 0 | 1 | 2 | 3 |
| **28g.** Store/Wateria | 0 | 1 | 2 | 3 |
| **28h.** Other (write in):  _________________ | 0 | 1 | 2 | 3 |

29. How frequently do you use the following sources for *drinking*?

**Use the following scale: 0= Never, 1= Rarely, 2= Frequently, 3= Always**

**[Note to interviewer: mark primary source]**

| **Water Source** | **Never** | **Rarely** | **Frequently** | **Always** |
| --- | --- | --- | --- | --- |
| **29a.** Public water system | 0 | 1 | 2 | 3 |
| **29b.** Bottled water from the store | 0 | 1 | 2 | 3 |
| **29c.** Privately owned well | 0 | 1 | 2 | 3 |
| **29d.** Delivered by a company | 0 | 1 | 2 | 3 |
| **29e.** Outdoor faucet or hose | 0 | 1 | 2 | 3 |
| **29f.** Vending machine | 0 | 1 | 2 | 3 |
| **29g.** Store/Wateria | 0 | 1 | 2 | 3 |
| **29h.** Other (write in):  _________________ | 0 | 1 | 2 | 3 |

30. How many times per day do you drink water by itself? For example, by itself means not mixed with Tang, Kool-Aid, coffee, or tea.

1= Once or less

2= 2-3 times

3= 4-5 times

4= More than 5 times

89= Don’t know

99= Refused

31. How much water do you drink at a time?

1 = 1 cup

2 = 2 cups

3 = 3 cups

4 = More than 4 cups (1 liter) _________________

89 = Don’t know

99 = Refused

32. How many times per day do you drink beverages prepared with cold water? For example, drinks like Tang and Kool-Aid.

0= Never 4= More than 5 times

1= Once or less 89= Don’t know

2= 2-3 times 99= Refused

3= 4-5 times

33. How many times per day do you drink beverages prepared with hot water? For example, drinks like coffee or tea.

0= Never

1= Once or less

2= 2-3 times

3= 4-5 times

4= More than 5 times

89= Don’t know

99= Refused

34. Do your close relatives or friends primarily drink tap water? **[If answer is “Yes”, please skip to question 36]**

0= No

1= Yes

89= Don’t Know

99= Refused

35. Do your close relatives or friends primarily drink bottled water or other sources of purchased water?

0= No

1= Yes

89= Don’t Know

99= Refused

36. What water source did you primarily drink as a child?

1= Tap

2= Bottled

3= Other _________________

89= Don’t know

99= Refused

**PART VI.**

***This section contains questions about different post water treatments***.

37. When you use **tap** water at home, how often do you **boil** it? **[If answer is “Never”, skip to question 39]**

0= Never 99= Refused

1= Rarely

2= Sometimes

3= Always

89= Don’t Know

38. Why do you boil it?

1= Taste

2= Health concerns

3= Hardness

4= Other _________________

89= Don’t Know

99= Refused

39. Do you think boiling water removes harmful **chemicals**?

0= No

1= Yes

89= Don’t Know

99= Refused

40. Do you think boiling water removes **microbes**?

0= No

1= Yes

89= Don’t Know

99= Refused

41. When you use **tap** water at home, how often do you **filter** it? **[If answer is “Never”, skip to question 44]**

0= Never

1= Rarely

2= Sometimes

3= Always

89= Don’t Know

99= Refused

42. Why do you filter your water?

1= Taste

2= Health concerns

3= Hardness

4= Other _________________

89= Don’t Know

99= Refused

43. What kind of water filter do you use? Mark all that apply.

1= Faucet filter

2= Pitcher with filter

3= Filter in fridge

4= Under the sink **[Note to interviewer, please specify the type]** _________________

5= Other _________________

89= Don’t know

99= Refused

44. Do you think filtering water removes harmful **chemicals**?

0= No

1= Yes

89= Don’t Know

99= Refused

45. Do you think filtering water removes **microbes**?

0= No

1= Yes

89= Don’t Know

99= Refused

46. When you use **tap** water at home, how often do you use **tablets or drops** to “clean” the water? [**If answer is “Never”, skip to question 48]**

0= Never

1= Rarely

2= Sometimes **[Note to interviewer, please specify which one]** _________________

3= Always **[Note to interviewer, please specify which one]** _________________

89= Don’t Know

99= Refused

47. Why do you treat your water with tablets or drops?

1= Taste

2= Health concerns

3= Hardness

4= Other _________________

89= Don’t Know

99= Refused

48. When you use **bottled** or other purchased water at home, how often do you **boil** it? **[If answer is “Never”, skip to question 50]**

0= Never

1= Rarely

2= Sometimes

3= Always

89= Don’t Know

99= Refused

49. Why do you **boil** it?

1= Taste

2= Health concerns

3= Hardness

4= Other _________________

89= Don’t Know

99= Refused

50. When you use **bottled** or other purchased water at home, how often do you **filter** it? **[If answer is “Never”, skip to question 53]**

0= Never

1= Rarely

2= Sometimes

3= Always

89= Don’t Know

99= Refused

51. Why do you **filter** it?

1= Taste

2= Health concerns

3= Hardness

4= Other _________________

89= Don’t Know

99= Refused

52. What kind of water filter do you use? Mark all that apply.

1= Faucet filter 99= Refused

2= Pitcher with filter

3= Filter in fridge

4= Under the sink **[Note to interviewer, please specify the type]** _________________

5= Other _________________

89= Don’t know

53. When you use **bottled** or other purchased water at home, how often do you use **tablets/drops** to “clean” the water? **[If answer is “Never”, skip to question 55]**

0= Never

1= Rarely

2= Sometimes **[Note to interviewer, please specify which one]** _________________

3= Always **[Note to interviewer, please specify which one]** _________________

89= Don’t Know

99= Refused

54. Why do you treat your water with tablets or drops?

1= Taste

2= Health concerns

3= Hardness

4= Other _________________

89= Don’t Know

99= Refused

55. If you knew that your tap water was safe to drink, would you drink it even if you didn’t like the taste?

0=No

1=Yes

89= Don’t know

99= Refused

**PART VII.**

***This section will cover questions about your water handling and water storage procedures.***

56. Do you reuse water containers? For example: gallon of water **[If answer is “No”, skip to question 61]**

0=No

1= No, I give them back to the water company

2=Yes

89= Don’t know

99= Refused

57. Do you clean the container in which you store your water? **[If answer is “No”, skip to question 60]**

0= No

1= Yes

89= Don’t know

99= Refused

58. How frequently do you clean the container?

1= Every couple of times I refill

2= Every time I refill

3= Other _________________

89= Don’t know

99= Refused

59. How do you clean the water container that you use to bring water to your home?

1= Rinse with water

2= Soap and water

3= Other ________________

89= Don’t know

99= Refused

60. For how long do you keep the water container before getting a new one?

1= 1 to 4 weeks

2= Longer than 1 month

3= Longer than 6 months

4= Longer than 1 year

89= Don’t know

99= Refused

61. Do you reuse disposable water bottles? **[If answer is “No”, skip to question 66]**

0= No

1= Yes

2= Sometimes

89= Don’t Know

99= Refused

62. Do you clean the disposable water bottles? **[If answer is “No”, skip to question 65]**

0= No

1= Yes

89= Don’t know

99= Refused

63. How frequently do you clean the disposable water bottle?

1= Every couple of times I refill 99= Refused

2= Every time I refill

3= Other _________________

89= Don’t know

64. How do you clean the disposable water bottle?

1= Rinse with water

2= Soap and Water

3= Other _________________

89= Don’t Know

99= Refused

65. For how long do you keep a disposable water bottle before throwing it out?

1= 1 day

2= 2 to 7 days

3= Longer than 1 week

89= Don’t know

99= Refused

66. When it is time to refill the water container or disposable water bottle, how do you transport it back?

1= I carry the container

2= Car

3= Bicycle

4= Motorcycle

5= Dolly

89= Don’t know

99= Refused

67. When you purchase water, do you immediately bring it back to your home? **[If answer is “Yes,” skip to question 70]**

0= No

1= Yes

2= Sometimes

89= Don’t Know

99= Refused

68. When you don’t bring it back home immediately, where do you keep it?

1= Car

2= In a building

3= Outside

89= Don’t know

99= Refused

69. For how long do you keep it there?

1= 1 hour

2= A couple of hours

3= Half a day

4= One day

5= Other _________________

89= Don’t know

99= Refused

70. Do you store your water in the refrigerator? **[If answer is “No”, skip to Part VIII]**

0= No

1= Yes

2= Sometimes

89= Don’t Know

99= Refused

71. Why do you refrigerate your water?

1= Tastes better

2= It is more refreshing

3= Health concerns

4= Other _________________

89= Don’t Know

99= Refused

72. What type of container do you use to store water in the refrigerator?

1= Plastic container in which it came in

2= Separate plastic container

3= Glass container

89= Don’t Know

99= Refused

**PART VIII.**

***The first few questions in this section will ask you about fluoride and fluoride supplements. Then you will be asked about your dental hygiene.***

73. Is fluoride important for your dental hygiene?

0= No

1= Yes

89= Don’t Know

99= Refused

**[Note to interviewer: Please read out loud the definition of fluoride provided below]**

*According to the Centers for Disease Control and Prevention, fluoride is a naturally occurring element that is proven to prevent, and even reverse, tooth decay. Fluoride helps re-mineralize the surface of your teeth and prevents cavities from forming.*

74. Does the water you drink contain fluoride?

0= No

1= Yes

89= Don’t Know

99= Refused

75. Have you ever been provided information about the importance of fluoride? **[If answer is “No”, skip to 77]**

0= No

1= Yes

89= Don’t Know

99= Refused

76. Who delivered this information? Mark all that apply.

1= School

2= Doctor

3= Dentist

4= Church

5= Nurse Practitioner (N.P.)

6= Physician Assistant (P.A.)

7= Mariposa Community Health Center

8= Community Health Worker (Promotora)

9= Other _________________

89= Don’t know

99= Refused

77. Have you ever received advice about using fluoride **supplements**? For example: toothpaste with fluoride, mouth rinse, gel, tablets.

0= No

1= Yes

89= Don’t know

99= Refused

78. Do you use fluoride supplements? For example: toothpaste with fluoride, mouth rinse, gel, tablets. **[If answer is “No” skip to 82]**

0= No

1= Yes

89= Don’t Know

99= Refused

79. What kind of fluoride supplements do you use? Mark all that apply.

1= Toothpaste with fluoride

2= Mouth rinse

3= Gel

4= Tablets

5= Other _________________

89= Don’t know

99= Refused

80. How often do you use fluoride supplements?

0= Never

1= Daily

2= Every other day

3= Weekly

4= Monthly

5= Yearly

89= Don’t know

99= Refused

81. How did you obtain the fluoride supplement?

1= Dentist gave me a sample

2= I purchased it

3= Someone gave it to me

4= Other _________________

89= Don’t know

99= Refused

82. Have you ever been treated for cavities? **[If answer is “No”, skip to question 84]**

0= No

1= Yes

89= Don’t Know

99= Refused

83. How many cavities have you been treated for?

1= 1

2= 2-5

3= More than 5 (specify) _________________

89= Don’t know

99= Refused

84. Have your children ever been treated for cavities?

0= No

1= Yes

89= Don’t know

99= Refused

85. Have your children received varnishing treatments from a doctor, dentist, Mariposa Community Health Center, Project Happy Smile, or their school? Check all that apply.

0= No

1= Doctor

2= Dentist

3= Mariposa Community Health Center

4= Project Happy Smile

5= School

6= Other _________________

89= Don’t know

99= Refused

**PART IX.**

***This section will cover questions about your interaction with medical and dental health providers.***

86. How often do you visit the dentist? [**If answer is “Never seen a dentist,” skip to question 89]**

1= Every 6 months

2=Yearly

3= Every 2 years

4= Never seen a dentist

5= Other _________________

89= Don’t know

99= Refused

87. Has a dentist recommended increasing your fluoride intake?

0= No 1= Yes 89= Don’t Know 99= Refused

88. Has a dentist talked to you about the type of water you drink?

0= No

1= Yes

89= Don’t know

99= Refused

**[Note to Interviewer: Ask the next question only if participant answered “Never seen a dentist” to question 86]**

89. What has prevented you from going to the dentist? Mark all that apply.

1= Time

2= Cost

3=Too far

4= Afraid of dentists

5= Not necessary to go to the dentist

6= Other _________________

89=Don’t know

99= Refused

90. How often do you visit a doctor or other medical provider? (Ex: Nurse Practitioner, Physicians Assistant) **[If answer is “Never see a medical care provider”, skip to question 92]**

1= Weekly

2= Monthly

3= Yearly

4= Every 2 years

5= Never see a medical care provider

6= Other _________________

89= Don’t know

99= Refused

91. Has a medical provider talked to you about the type of water you drink?

0= No

1= Yes

89= Don’t Know

99= Refused

**[Note to Interviewer: Ask the next question only if participant answered “Never see a medical care provider” to question 90]**

92. What has prevented you from seeing a medical care provider?

1= Time 4= Afraid of doctors 89= Don’t know

2= Cost 5= Not necessary to go to medical care provider 99= Refused

3= Too far 6= Other _________________

**PART X.**

93. [Interviewer write in] Gender: ___________________________

94. What is your age? ___________________________

95. What is your ethnicity? ___________________________

96. Where were you born? ___________________________

97. For how many years have you lived in Nogales, AZ? ___________________________

98. For how many years have you lived in Nogales, Sonora, MX? _______________________

99. What is the highest grade of school that you have completed?

0= No school

Elementary School High School Technical School College (for credit only)

1= 1st grade 9= 9th grade 13= 1 year 17= 1 year

2= 2nd grade 10= 10th grade 14= 2 years 18= 2 years

3= 3rd grade 11= 11th grade 15= 3 years 19= 3 years

4= 4th grade 12= 12th grade 16= 4 years 20= 4 years

5= 5th grade 21= 5+ years

6= 6th grade

7= 7th grade

8= 8th grade

89= Don't know

99= Refused

100. What is the highest degree received: ___________________________

101. Please indicate your household yearly income.

**[Interviewer: Calculate yearly income if they give you weekly or monthly income]**

0= less than $5,999 5= $26,000-$29,999 99= Refused

1= $6,000-$9,999 6= $30,000-$34,999

2= $10,000-$15,999 7= $35,000-$40,000

3= $16,000-$20,999 8= more than $40,000

4= $21,000-$25,999 89= Don’t Know

102. How many adults live in this home? ________________________

89= Don’t know

99=Refused

103. How many children live in this home? ___________________________

89= Don’t know

99= Refused

104. What are the ages of the children? ___________________________

89= Don’t know

99= Refused

105. Are you currently working, unemployed and/or looking for work, or on layoff from a job, retired, going to school, keeping house, or something else?

1= Working full time (30 or more hours per week)

2= Working part time (fewer than 30 hours per week)

3= Looking for work

4= Retired

5= Going to school

6= Keeping house

7= Something else (specify) __________________

89= Don't know

99= Refused

106. What is your occupation? ___________________________

******************************************************************************

**[Interviewer] At the end of the interview thank the participant, you may say:**

### Do you have any questions? If you have any other questions later, you may call us at (520) 626-0006. Thank you for participating in our study. The information obtained will be very valuable. Sorry for any inconvenience which we may have caused. Once again, thank you for your time.
